# Risk estimation for stroke-associated pneumonia in acute ischemic stroke: a nomogram-based approach

**DOI:** 10.1055/s-0046-1820531

**Published:** 2026-05-18

**Authors:** Jiao Wang, Shuai Zhang, Yueguang Cheng, Bing Yan

**Affiliations:** 1 Beijing Jingmei Group General Hospital, Department of Emergency Medicine, Beijing, China.; 2 Aimei Medical Aesthetic Clinic, Department of Plastic Surgery, Qiqihar, Heilongjiang, China.

**Keywords:** Ischemic Stroke, Pneumonia, Nomograms

## Abstract

**Background:**

Stroke-associated pneumonia (SAP) is a common complication in patients with acute ischemic stroke (AIS), leading to higher mortality and poor functional outcomes. Early identification of at-risk individuals is critical for timely interventions.

**Objective:**

To identify the independent risk factors for SAP in AIS patients and to develop a predictive nomogram for early risk stratification.

**Methods:**

We conducted a retrospective analysis of 280 AIS patients admitted between January 2021 and August 2025. Multivariable logistic regression identified independent SAP risk factors, and a nomogram was developed. Model performance was evaluated through receiver operating characteristic (ROC) curve analysis, calibration plots, and decision curve analysis (DCA). Internal validation was performed using bootstrap resampling (1 thousand iterations).

**Results:**

The incidence of SAP was of 30.0% (84/280). The independent risk factors included advanced age, atrial fibrillation, higher score on the National Institutes of Health Stroke Scale (NIHSS), nasogastric tube insertion, mechanical ventilation, and elevated monocyte-to-lymphocyte ratio (MLR). Higher albumin levels were protective. The nomogram showed good discrimination (area under the curve [AUC]= 0.816; 95%CI: 0.765–0.871), satisfactory calibration, and favorable clinical usefulness, as demonstrated by the DCA.

**Conclusion:**

We developed a nomogram to predict SAP risk in AIS patients. The key risk factors included advanced age, atrial fibrillation, NIHSS score, nasogastric tube insertion, mechanical ventilation, and elevated MLR, while higher albumin levels were protective. The nomogram demonstrated strong discriminatory power and clinical usefulness, supporting early risk stratification and targeted interventions.

## INTRODUCTION

Acute ischemic stroke (AIS) is a critical cause of disability and mortality around the world, imposing a heavy burden on patients, their families, and healthcare infrastructures. 1 2 In individuals diagnosed with AIS, stroke-associated pneumonia (SAP) occurs frequently and is considered a serious medical condition, as it prolongs hospitalization, increases the risk of death, and worsens long-term outcomes. 3 4 5 The prompt detection of individuals at high risk of developing SAP plays a key role in initiating early preventive measures and achieving better outcomes.

The pathogenesis of SAP is multifactorial, involving stroke-induced immune dysregulation, impaired neurological function, and enhanced susceptibility of the pulmonary microenvironment to bacterial colonization. 6 7 8 Previous research has identified advanced age, more severe strokes, coexisting medical conditions, and abnormal laboratory findings as key risk factors. 6 7 8 9 10 Nevertheless, reliance on single predictors has limited clinical applicability, and robust tools that integrate multiple variables to improve risk stratification remain urgently needed.

Nomograms, which translate multivariable regression models into a user-friendly visual tool, have gained wide application in oncology, cardiovascular disease, and neurological disorders to enhance individualized prediction. 11 12 Despite existing research, there is a continued absence of validated predictive models for SAP in AIS patients. The purpose of the current study was to determine the independent predictors of SAP and to develop a nomogram model designed to equip clinicians with a dependable and practical tool for early risk evaluation and individualized management in AIS.

## METHODS

### Study population and design

We conducted a retrospective analysis involving 280 AIS patients admitted to the Emergency Department of the Beijing Jingmei Group General Hospital between January 1, 2021, and August 31, 2025. Acute ischemic stroke was diagnosed in all patients based on the criteria of the World Health Organization (WHO) and the Chinese Stroke Diagnosis and Treatment Guidelines, with confirmation by cranial computed tomography (CT) or magnetic resonance imaging (MRI) scans. The inclusion criteria consisted of: age ≥18 years; admission within 7 days of symptom onset; and availability of complete clinical and laboratory data. The exclusion criteria were: preadmission pulmonary infection; comorbidities such as malignant tumors, severe heart, liver, or kidney dysfunction; immune deficiencies or long-term immunosuppressive therapy; incomplete data; and presence of severe intracerebral hemorrhage or other serious comorbidities that may affect the diagnosis or treatment of stroke.

Approval was granted by the Ethics Committee of the Beijing Jingmei Group General Hospital, and all procedures followed the Declaration of Helsinki and applicable ethical principles. All data were anonymized.

### Data analysis

The continuous variables were reported as mean ± standard deviation or median (interquartile range, IQR) values following normality tests. Differences between groups were analyzed using *t* -tests or Mann-Whitney U tests, as applicable. For the categorical variables, the results were presented as frequencies and percentages, and statistical differences were assessed with Chi-squared (χ ^2^ ) or Fisher's exact tests. Those factors identified as significant in the univariate level were further analyzed using multivariable logistic regression to estimate odds ratios (ORs) and 95%CIs A nomogram for SAP prediction was generated in the R (R Foundation for Statistical Computing) software, version 4.3.1, based on the regression model. Its discriminatory ability, calibration, and clinical usefulness were examined through a receiver operating characteristic (ROC) curve, calibration plots, and decision curve analysis (DCA) respectively. To limit overfitting, internal validation was performed using 1 thousand bootstrap resamples. Statistical analyses were carried out in the R software, with significance determined by a two-sided *p* -value < 0.05.

## RESULTS

### Baseline demographic and clinical characteristics

A total of 280 patients with AIS were enrolled, comprising 84 subjects (30.0%) in the SAP group and 196 subjects (70.0%) in the non-SAP group ( Table 1 ). Patients who developed SAP were significantly older compared to the non-SAP group (72.4 ± 11.0 versus. 66.5 ± 11.8 years; *p* < 0.001) and had higher scores on the score on the National Institutes of Health Stroke Scale (NIHSS) (11 [6–18] versus 5 [3–9]; *p* < 0.001). In terms of comorbidities, the proportion of atrial fibrillation was significantly higher in the SAP group compared to the non-SAP group (24.1% versus 12.8%; *p* = 0.033), while no significant differences were found regarding hypertension, diabetes, or coronary artery disease. Regarding treatment, the SAP group had significantly higher rates of nasogastric tube insertion (46.4% versus 12.8%; *p* < 0.001) and mechanical ventilation (17.9% versus 2.6%; *p* < 0.001) compared to the non-SAP group. Laboratory results showed that the SAP group had lower albumin levels (35.0 ± 4.2 g/L versus 37.7 ± 3.6 g/L; *p* < 0.001), higher white blood cell counts (9.3 ± 3.1 × 10 ^9^ /L versus 7.6 ± 2.4 × 10 ^9^ /L; *p* = 0.003), higher monocyte-to-lymphocyte ratios (MLRs; 0.44 [0.34–0.67] versus 0.28 [0.21–0.36] ; *p* < 0.001), and lower serum iron levels (13.0 ± 4.5 µmol/L versus 14.8 ± 4.2 µmol/L; *p* = 0.035) comparted to the non-SAP group.

**Table 1:** Baseline characteristics of patients with and without SAP (N = 280)

Category	Variable	Non-SAP (n = 196)	SAP (n = 84)	*p* -value
Demographics	Mean age (years)	66.5 ± 11.8	72.4 ± 11.0	< 0.001
	Male sex: n (%)	120 (61.2)	56 (66.7)	0.466
	Mean BMI (kg/m ^2^ )	24.9 ± 3.8	25.4 ± 4.1	0.400
Comorbidities	Hypertension: n (%)	134 (68.4)	55 (65.5)	0.738
	Diabetes mellitus: n (%)	50 (25.5)	25 (29.8)	0.556
	Atrial fibrillation: n (%)	25 (12.8)	20 (24.1)	0.033
	Heart failure: n (%)	6 (3.1)	7 (8.3)	0.107
	Coronary artery disease: n (%)	20 (10.2)	10 (12.0)	0.833
	Smoking history: n (%)	72 (36.7)	28 (33.3)	0.683
	Drinking history: n (%)	61 (31.1)	23 (27.4)	0.629
Clinical features	NIHSS score: median (IQR)	5 (3–9)	11 (6–18)	< 0.001
	TOAST – LAA: n (%)	51 (26.0)	21 (25.0)	0.976
	Stroke territory: anterior circulation: n (%)	135 (68.9)	63 (75.0)	0.374
	Onset-to-admission time (hours): median (IQR)	12 (6–24)	14 (8–30)	0.180
Treatment	Nasogastric tube: n (%)	25 (12.8)	39 (46.4)	< 0.001
	Mechanical ventilation: n (%)	5 (2.6)	15 (17.9)	< 0.001
	Acid-suppressant use: n (%)	117 (59.7)	62 (73.8)	0.034
Laboratory findings	Mean LDL (mmol/L)	2.8 ± 0.7	2.6 ± 0.8	0.060
	Mean albumin (g/L)	37.7 ± 3.6	35.0 ± 4.2	< 0.001
	Mean WBC (×10 ^9^ /L)	7.6 ± 2.4	9.3 ± 3.1	0.003
	Mean RBC (×10 ^12^ /L)	4.4 ± 0.5	4.3 ± 0.6	0.090
	Mean Hemoglobin (g/L)	132.5 ± 15.8	129.0 ± 17.2	0.084
	Mean platelet (×10 ^9^ /L)	210.0 ± 61.0	188.0 ± 66.0	0.062
	MLR: median (IQR)	0.28 (0.21–0.36)	0.44 (0.34–0.67)	< 0.001
	Mean serum iron (µmol/L)	14.8 ± 4.2	13.0 ± 4.5	0.035

Abbreviations: BMI, body mass index; IQR, interquartile range (first–third quartiles); LAA, large-artery atherosclerosis; LDL, low-density lipoprotein; MLR, monocyte-to-lymphocyte ratio; NIHSS, National Institutes of Health Stroke Scale; RBC, red blood cell; SAP, stroke-associated pneumonia; TOAST, Trial of Org 10172 in Acute Stroke Treatment; WBC, white blood cell.

### Multivariable logistic regression analysis ( Table 2 )

**Table 2:** Multivariate logistic regression analysis of risk the factors for SAP

Variable	OR	95%CI	*p* -value
Age	1.02	1.01–1.04	0.006
Atrial fibrillation	2.00	1.15–3.48	0.014
NIHSS score	1.12	1.08–1.17	< 0.001
Nasogastric tube	4.00	2.10–7.62	< 0.001
Mechanical ventilation	6.00	2.10–17.2	< 0.001
Acid-suppressant use	1.30	0.86–1.98	0.212
Albumin	0.95	0.92–0.98	0.002
MLR	1.20	1.10–1.32	< 0.001
Serum iron	0.98	0.95–1.01	0.188

Abbreviations: MLR, monocyte-to-lymphocyte ratio; NIHSS, National Institutes of Health Stroke Scale; OR, odds ratio; SAP, stroke-associated pneumonia.

We included variables that reached statistical significance in the univariate analysis for further evaluation using a multivariable logistic regression model. Due to the strong correlation between white blood cell count and the MLR, the white blood cell count was excluded from the multivariable regression model to avoid multicollinearity. The multivariable analysis results showed that age (OR = 1.02; 95%CI: 1.01–1.04; *p* = 0.006), atrial fibrillation (OR = 2.00; 95%CI: 1.15–3.48; *p* = 0.014), NIHSS score (OR = 1.12; 95%CI: 1.08–1.17; *p* < 0.001), nasogastric tube insertion (OR = 4.00; 95%CI: 2.10–7.62; *p* < 0.001), mechanical ventilation (OR = 6.00; 95%CI: 2.10–17.2; *p* < 0.001), and MLR (OR = 1.20; 95%CI: 1.10–1.32; *p* < 0.001) were independent risk factors for SAP, while albumin (OR = 0.95; 95%CI: 0.92–0.98; *p* = 0.002) was identified as a protective factor.

### Establishment and validation of the predictive nomogram

**Figure 1:**
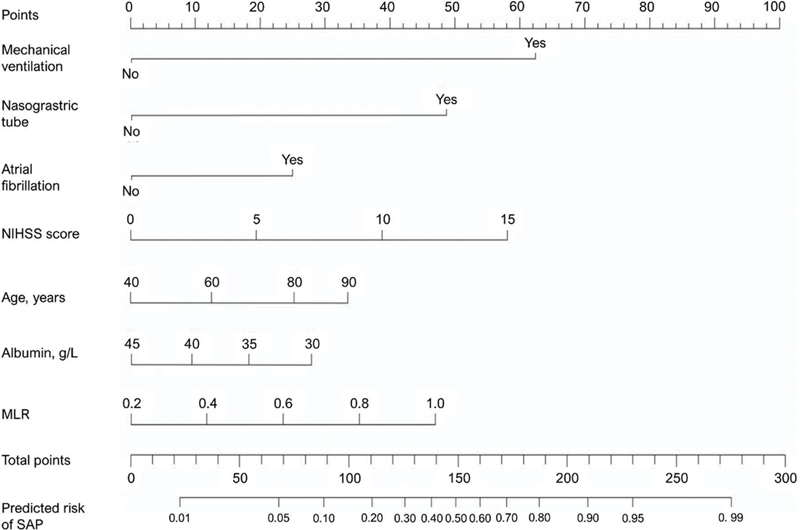
Nomogram prediction model for acute ischemic stroke complicated by pneumonia.

**Figure 2:**
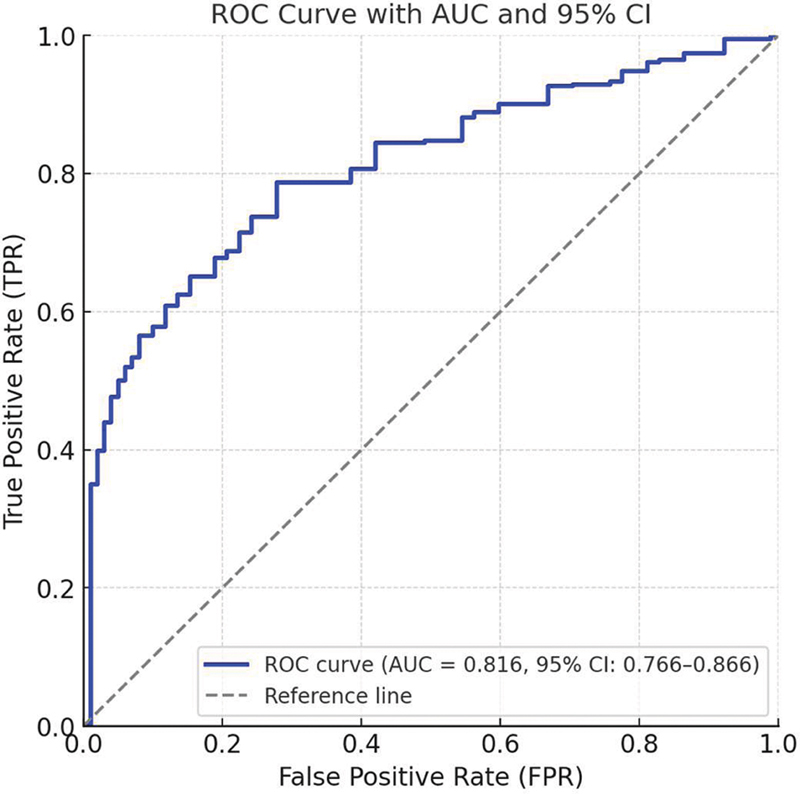
Receiver operating characteristic (ROC) curves of the predictive nomogram.

**Figure 3:**
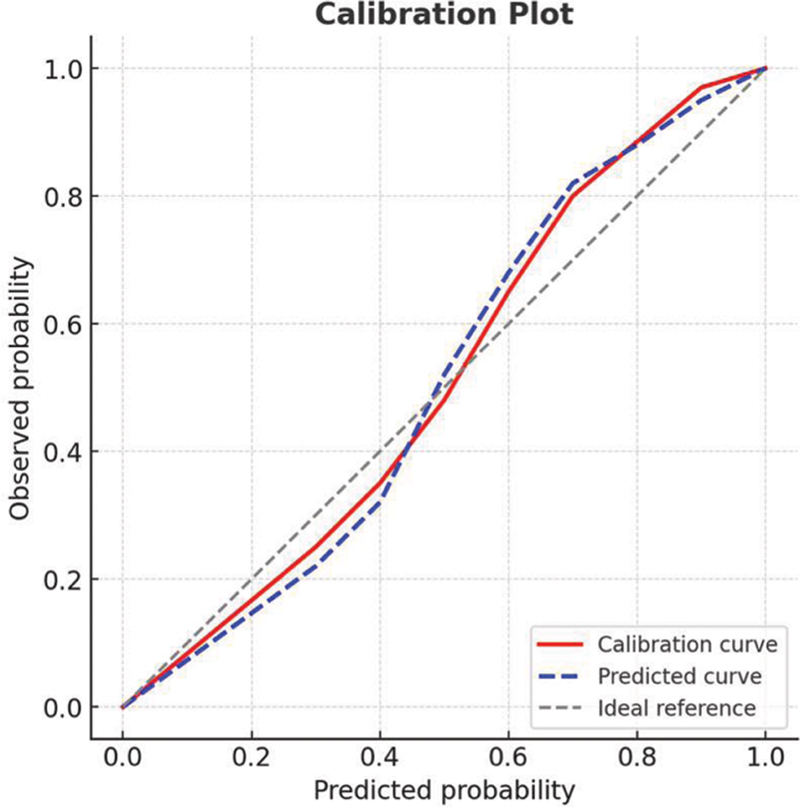
Calibration curves of the predictive nomogram.

**Figure 4:**
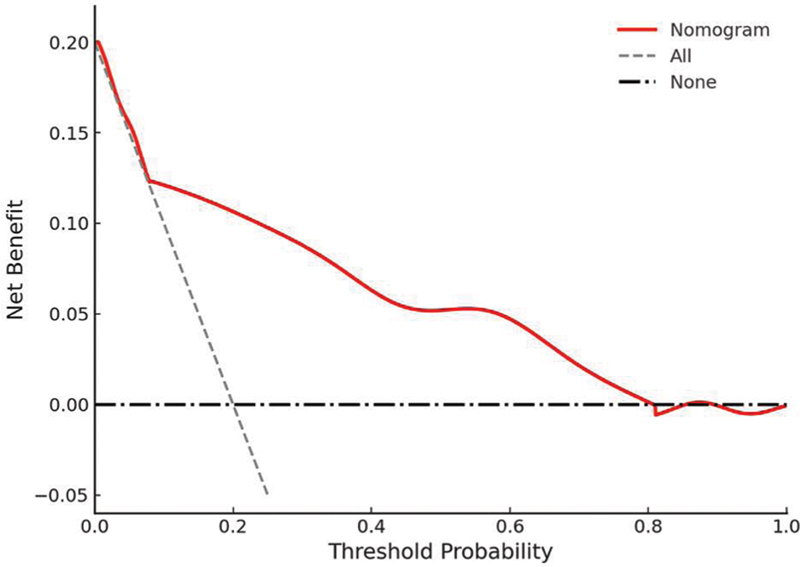
Decision curve analysis of the predictive nomogram.

A nomogram to predict SAP was developed based on the variables identified in the multivariable logistic regression analysis ( Figure 1 ). The variables included in the model were age, atrial fibrillation, NIHSS score, use of a nasogastric tube, mechanical ventilation, albumin, and MLR. Each predictor corresponds to a specific score, and the total score indicates the risk of developing SAP, with higher scores representing greater risk. The predictive performance of the nomogram was supported by internal validation. An area under the curve (AUC) of 0.816 (95%CI: 0.765–0.871) was obtained from the ROC curve analysis ( Figure 2 ), demonstrating robust discrimination. The calibration analysis ( Figure 3 ) further indicated reliable alignment of predicted probabilities with observed outcomes. The DCA ( Figure 4 ) revealed that, within a threshold probability range of 9 to 81%, the nomogram model provided a higher net benefit than the *treatment for all* and *no treatment* strategies, highlighting its clinical applicability.

## DISCUSSION

### Main findings

The current study included 280 patients with AIS; the incidence of SAP was of 30.0%. Through multivariable logistic regression analysis, we identified age, atrial fibrillation, NIHSS score, nasogastric tube insertion, mechanical ventilation, and an elevated MLR as independent risk factors for the development of SAP. In contrast, elevated albumin levels appeared to exert a protective effect. These selected factors were used to develop a nomogram predicting SAP risk in AIS patients. The ROC curve showed good discrimination (AUC = 0.816), the calibration curve demonstrated satisfactory calibration, and the DCA indicated significant clinical usefulness of the model.

### Association between risk factors and SAP

#### *Age and SAP*

Age, as an important risk factor for the development of SAP, 10 13 was also confirmed in the present study. Among patients with AIS, older individuals may experience immune aging, which can lead to functional disruptions, such as cytokine imbalance, increasing their susceptibility to SAP. Moreover, immune aging not only weakens cellular immune responses but may also trigger chronic low-grade inflammation, further reducing the elderly's ability to resist infections. 14 15 16 17 Additionally, older adults often present multiple comorbidities, such as hypertension, diabetes, and cardiovascular diseases, which can further increase the risk of developing SAP.

#### *Atrial fibrillation and SAP*

The current study found that atrial fibrillation was associated with higher risk of developing SAP, a result consistent with the findings of previous studies. 18 19 20 As reported by Li and He, 19 the results of the logistic regression analysis showed that atrial fibrillation (OR = 3.585; 95%CI: 1.605–8.007; *p* = 0.019) was an independent risk factor associated with SAP. A study conducted by Kuo et al. 20 included 546 patients, and their multivariate logistic regression analysis also identified atrial fibrillation as a major predictor of SAP. Atrial fibrillation leads to abnormalities in heart structure and function, causing hemodynamic changes and the formation of atrial thrombi, which increases the risk of multiple intracranial strokes. This, in turn, enhances poststroke immune suppression, making patients more susceptible to SAP. 21 22 23 In addition, in patients with atrial fibrillation, frequent episodes of this condition and irregular heartbeats may increase airway instability or make respiratory symptoms, such as coughing, shortness of breath, and sleep apnea, more likely to occur, thereby potentially increasing the risk of developing SAP. 24 25

#### *Stroke severity (NIHSS score) and SAP*

A higher NIHSS score indicates greater stroke severity. The current study revealed that, for every 1-point increase in the NIHSS score, the likelihood of developing SAP rose by about 12%. This result aligns with those of several international studies. 3 9 26 27 It suggests that more severe strokes increase the likelihood of swallowing difficulties, impaired consciousness, and respiratory dysfunction, thereby elevating the risk of pneumonia.

#### *Clinical intervention factors (nasogastric tube insertion, mechanical ventilation) and SAP*

Placement of a nasogastric tube and use of mechanical ventilation were associated with the risk of developing SAP, as reported in certain studies. 6 27 28 29 Studies 30 have also shown that mechanical ventilation and nasogastric tube insertion can promote the colonization of bacteria in the oropharynx, increasing the likelihood of aspiration, which subsequently leads to pneumonia. Therefore, clinicians must carefully consider the indications for these interventions and strengthen nursing management. It should also be noted that mechanical ventilation itself is used as a treatment when pneumonia progresses to a severe stage, indicating a bidirectional relationship between mechanical ventilation as a risk factor and pneumonia as an outcome.

### Laboratory indicators (albumin, MLR) and SAP

The present study found a positive correlation involving hypoalbuminemia, elevated MLR, and the occurrence of SAP. Our findings correspond to those of some previous reports. 31 32 33 34 35

Low albumin levels are typically associated with malnutrition and immune suppression, which can reduce the patients' immune response, making them more susceptible to infections. Additionally, low albumin levels may enhance oxidative stress and inflammation, further increasing the risk of developing SAP. 32 34 The MLR is an indicator of inflammation. Elevated MLR typically signifies an overactive immune response or an immune-suppression state, and this immune dysregulation leads to enhanced inflammatory responses, which, in turn, increase the risk of developing SAP. 35 36

### Evaluation of the predictive model's performance

The nomogram model developed in the current study underwent internal validation and demonstrated strong performance. The ROC curve analysis revealed an AUC of 0.816, indicating high discriminatory ability. The calibration curve indicated strong concordance between the model-predicted risk and the observed risk. Furthermore, the DCA demonstrated that, within a threshold probability range of 9 to 81%, the model provided higher net benefits compared to the *treatment for all* and *no treatment* strategies, further confirming its clinical applicability.

### Clinical significance

The risk prediction nomogram developed in the present study is intuitive and practical, enabling clinicians to evaluate the SAP risk among patients with AIS. Through personalized risk assessments, clinicians can implement early interventions such as enhanced swallowing function assessments, better nutritional support, oral care, and airway management, which may lower the risk of SAP and improve patient outcomes.

### Limitations and future directions

There are several limitations to the current study. First, it was a retrospective analysis conducted at a single center, with a relatively small sample size. Selection bias may have been unavoidable. Second, the model was only internally validated and lacked external validation; future studies are needed to test its generalizability in large, multicenter, prospective cohorts. In addition, the study did not account for potential factors such as dynamic changes in poststroke immune function or microbiological test results, which may have led to an underestimation of the impact of certain variables. To confirm its generalizability, future studies should examine this model in larger, multicenter, prospective cohorts. Moreover, incorporating inflammatory biomarkers, immune indicators, imaging data, and artificial intelligence techniques may further improve its predictive accuracy. Integrating risk prediction with clinical-intervention pathways could help translate prediction into effective risk management, ultimately guiding treatment decisions and improving the outcomes of stroke patients. Furthermore, swallowing dysfunction is a common complication in patients with AIS, and it is considered a significant risk factor for the development of SAP. Swallowing disorders can increase the risk of aspiration, particularly in patients with nasogastric tube insertion or mechanical ventilation, thereby promoting bacterial colonization in the lungs. However, the present study did not quantify or systematically assess swallowing function in patients; as such, swallowing dysfunction was not included as a risk factor in our analysis model. Nevertheless, swallowing dysfunction remains a potential high-risk factor for the development of SAP, and we recommend that future studies further explore this relationship, using standardized and objective assessment tools (such as the videofluoroscopic swallowing study) to more accurately quantify swallowing function and clarify its specific impact on the risk of developing SAP.

In conclusion, in the current study, we developed a nomogram to predict SAP risk in AIS patients. The key independent risk factors included advanced age, atrial fibrillation, NIHSS score, nasogastric tube insertion, mechanical ventilation, and elevated MLR, while higher albumin levels were protective. The nomogram exhibited encouraging discriminatory ability and clinical usefulness, offering potential support for early risk stratification and targeted interventions.
